# In vitro production of two chitinolytic proteins with an inhibiting effect on the insect coffee berry borer, *Hypothenemus hampei *(Ferrari) (Coleoptera: Curculionidae) and the fungus *Hemileia vastatrix *the most limiting pests of coffee crops

**DOI:** 10.1186/2191-0855-2-22

**Published:** 2012-03-30

**Authors:** Claudia P Martínez, Claudia Echeverri, Juan C Florez, Alvaro L Gaitan, Carmenza E Góngora

**Affiliations:** 1 Entomology Department, Cenicafé, Federacafe (National Coffee Research Centre - National Federation of Coffee Growers), PlanAlto Km 4 via antigua a Manizales, Chinchina, Caldas, Colombia; 2 Plant Pathology Department, Cenicafé, Federacafe (National Coffee Research Centre - National Federation of Coffee Growers), PlanAlto Km 4 via antigua a Manizales, Chinchina, Caldas, Colombia

**Keywords:** Coffee-based artificial diet, Uredinospores, Arrested development, Fungal cell wall, Growth, Mortality

## Abstract

Two genes from *Streptomyces albidoflavus*, one exochitinase (905-bp) and an endochitinase (1100-bp) were functionally expressed in *Escherichia coli *in form of a fusion protein with a maltose binding protein (MBP). The goal was to produce and test proteins that inhibit both the coffee berry borer insect *Hypothenemus hampei *and the coffee rust fungus *Hemileia vastatrix*. Both recombinant proteins MBP/exochitinase and MBP/endochitinase showed chitinolytic activity. When recombinant purified proteins were added to an artificial coffee-based diet for the coffee berry borer, MBP/exochitinase at a concentration of 0.5% W/W caused delayed growth of larvae and 100% mortality between days 8 and 15, while MBP/endochitinase caused 100% mortality at day 35. *H. vastatrix *urediniospores presented total cell wall degradation in their germinative tubes within 18 h of exposure to the proteins at enzyme concentrations of 5 and 6 mg ml^-1^, with exochitinase having the greatest effect. The dual deleterious effect of *S. albidoflavus *chitinases on two of the most limiting coffee pests worldwide, the coffee borer and the coffee rust, make them potential elements to be incorporated in integrated control strategies.

## Introduction

Colombia is one of the most important countries for production of mild coffee (*Coffea arabica *L.), with over 870,000 Ha planted (Federacafé 2010). The coffee berry borer, *Hypothenemus hampei *(Ferrari) (Coleoptera: Curculionidae: Scolytidae), is the most significant pest of the Colombian coffee crop, and it is found in all the coffee-growing regions of the world (Benavides et al. 2012; Bustillo 2002). Female insects fly towards coffee beans and bore into them until reaching the seed endocarpium, where they deposit their eggs. After the eggs hatch, larvae feed on the seed, causing weight loss in the grain, decreasing quality due to fungal contamination, and the falling of small cherries to the ground (Duque et al. 1997).

The coffee leaf rust, caused by the fungus *Hemileia vastatrix *Berk. and Br. (Uredinales) is the main limiting disease of susceptible *Coffea arabica *varieties around the world. Although all its life cycle occurs in the leaves, cholorosis and defoliation affect the filling and maturation of coffee beans, reducing bean size and quality. Several rust resistant varieties have been bred in various coffee producing countries, including the Colombia and Castillo varieties (Castillo and Moreno 1988; Alvarado et al. 2005) but the ability of *H. vastatrix *to overcome such genetic resistance threatens continuously the durability of this control method. To date, the natural resistance to the coffee borer identified in *C. arabica *germplasm collections is considered weak, and sources of resistance genes against the coffee leaf rust are limited.

Both parasites, the coffee berry borer and the coffee rust, contain chitin, an insoluble linear β-1,4 polymer of N-acetyl β-D- glucosamide, that is a structural component present in the cuticle and shells of arthropods and mollusks, in the cell wall of fungi and some algae, and in certain nematode stages (Brydon et al. 1989; Elango et al. 1982; Gooday 1990).

In insects, chitin is a significant component of the cuticle, which constitutes part of the exoskeleton, and at least partially lines along internal organs including the digestive tract; in addition it is a structural component of the peritrophic membrane that covers the midgut (Peters 1992). In fungi, α-chitin is an essential component of the structure of the cell wall (Gooday et al. 1992).

Chitin can be enzymatically cleaved by two main enzyme classes: chitinases and N-acetyl-β-D-glucosaminidase. Chitinases (E.C. 3.2.1.14) catalyze the hydrolysis of the linear polymer β-1,4 linkages in chitin and chitooligomers resulting in the release of short chitooligomers. Depending on their cleavage pattern chitinases can be divided in endochitinases and exochitinases. Endochitinases degrade chitin from any point along the polymer chain forming random size length products while exochitinases cleave from the non-reduced end and the released product is (GLcNac)2 (Seidl 2008). Fungal chitinases can be divided into three different subgroups, namely, A, B and C, based on the amino acid sequences of their GH 18 modules. These subgroups differ in the architectures of their substrate-binding cleft and, thus, their enzymatic activities (exo vs. endo) and also contain different carbohydrate-binding modules (Gruber et al. 2011; Seidl 2008). Horn et al. (2006) studying the chitinolytic system of the bacterium *Serratia marcescens *demonstrated another way to classify the enzymatic properties of chitinases by grouping them into processive and non-processive enzymes. Processive chitinases do not release the substrate after hydrolytic cleavage but slide it through the active site-tunnel for the next cleavage step to occur. The presence of a carbohydrate binding domain can enhance processivity, but is not essential for it. Non-processive chitinases dissociate completely from the substrate after hydrolysis.

Chitinases are essential to arthropods (Chen 1987) and fungi (Review Seidl 2008; Hartl et al. 2011) to regulate the presence of chitin during growth, development, and differentiation. In insects, chitinases are involved in molting and digestion processes (see review Muthukrishnan et al. 2011), while in fungi, they mainly help to degrade and mobilize organic matter and to interfere with the growth of other fungi.

Cuticle in insects is the target site of chitinolytic enzymes and its damage affects survival (Hegedus et al. 2009). Alterations of the peritrophic membrane disturb its selective permeability properties resulting in nutritional imbalances, by affecting food acquisition, water retention or excretion and digestion, besides increasing vulnerability against abrasion by food particles or invasion by pathogens (Peters 1992), and reducing protection from toxic compounds (Barbehenn and Constabel 2011). In plants, evidence of the role of chitinolytic enzymes as chemical defense agents against fungi is corroborated by the coordinated induction of those enzymes in response to pathogen invasion (Gooday et al. 1992; Ma et al. 2009), the inhibition of fungal spore germination and mycelial growth, and their ability to hydrolyze fungal cell walls (Arlorio et al. 1992; Schlumbaum et al. 1986). The expression of higher levels of those enzymes in resistant cultivars compared to susceptible cultivars (Hughes and Dickerson 1991) and the resistance enhancement to fungal pathogens resulting from the introduction into plants of several cloned plant and microbial chitinasases (see Schickler and Chet 1997 for a review) demonstrate the role of these enzymes in plant defense.

A chitinase mixture from *Streptomyces albidoflavus *(NRRL B-16746 Agricultural Research Service Culture Collection, United States Department of Agriculture, Peoria, IL) containing three endochitinases, two 1,4-β-D-chitobiosidases (exochitinase) and one glucosaminidase, with ability to degrade chitin under a wide pH range, significantly reduced the germination, growth and mycelia development of plant pathogenic fungi such as *Botrytis cinerea *and *Fusarium oxysporum *(Broadway et al. 1995). In addition, the mixture affected the growth, development and/or survival of the budworm *Heliothis virescens*, the looper *Trichoplusia ni*, the whitefly *Bemisia argentifolii*, the potato aphid *Myzus persicae*, and the coffee berry borer *H. hampei *(Broadway et al. 1998).

Góngora et al. (2001) cloned the coding sequences of two enzymes of the *S. albidoflavus *mixture: an endochitinase and a chitibiosidase (exochitinase) (Gongora 1999; Broadway and Harman 2000) and transformed tomato plants (*Lycopersicon esculentum*) with these genes. The plants showed effect on the growth and development of *Trichoplusia ni *larvae by consistently decreasing the growth rate of the insect and altering the chitin of the intestinal peritrophic membrane; thereby increasing the permeability of the membrane. Likewise, other authors have identified the inhibitory effects of plant chitinases on insects. Lawrence and Novak (2006) reported the over-expression of a poplar chitinase in tomato plants resulting in an arresting effect on the Colorado potato beetle. Similarly, the induction of chitinases in plants as a response to insect caused stress has been reported (Lippmann et al. 2009). Chitinases isolated from insects and over-expressed in plants can also be effective insect resistance factors (Ding et al. 1998; Kramer and Muthukrishnan 1997).

To determine if the endochitinase and exochitinase proteins isolated from *S. albidoflavus *reported by Góngora et al. (2001) were simultaneously effective against the coffee berry borer and the coffee leaf rust, both genes were expressed in the pMAL Protein Fusion and Purification System, and the recombinant proteins produced were tested in vivo by adding them to either artificial coffee-based diets or to germinating *H. vastatrix *urediniospores. Significant biological activity of the proteins will promote them as prospective candidates for expression in coffee plants to confer new insect and pathogen resistance in coffee.

## Materials and methods

### Cloning of the exochitinase and endochitinase coding sequences in the pMAL-c2X vector

A 905-bp cDNA of the exochitinase gene and a 1.100-bp endochitinase gene from *S. albidoflavus *NRRL B-16746 reported by Gongora (1999) and in the US Patent 6,069,299 (Broadway and Harman 2000) were cloned from a pGEM^®^T-Easy construct as a *Bam*H I-*Hin*d III fragment into the pMAL-cX2 vector (New England Biolabs, Beverly, MA, USA) (New England Biolabs 2003), to produce a maltose-binding protein (MBP) fusion whose component proteins can be cleaved apart with the specific protease Factor Xa (New England Biolabs). The resulting plasmids pMAL-c2-exochitinase and pMAL-c2-endochitinase were transformed into JM109 *E. coli*, followed by sequencing confirmation of in-frame gene insertion into the pMAl-cX2 plasmid.

The DNA and protein sequences (supplementary material) of both genes were analyzed using the Basic Local Alignment Search Tool (BLAST) from the NCBI database and the Uniprot database http://www.uniprot.org.

### Expression and production of the MBP/exochitinase and MBP/endochitinase

Cells transformed with the expression vector pMAL-c2- exochitinase or pMAL-c2-endochitinase were cultivated at 170 rpm and 37°C for 2-3 h until reaching an absorbance of ~0.5-0.6 on liquid broth (LB) medium and ampicillin, and then induced by adding isopropylthiogalactoside to a final concentration of 0.3 mM. The cultures were grown at 37°C for 3 h. Then, they were centrifuged at 4,500 rpm for 15 min at 4°C, the pellet was collected and cellular proteins were separated from the inclusion bodies by sonication at amplitude of 60% at 30-sec intervals over 2.5 minutes. Protein concentration was determined for each sample using the Bio-Rad Protein Assay following the Bradford dye-binding procedure (Bio-Rad. Richmond, CA, USA), a 20 μL of the induced sample was taken, along with 80 μL of NaCl and 2.5 mL of Bradford's reagent. The samples were read in a spectrophotometer (Unicam UV/VIS) at 595 nm, yielded concentrations of 9-11 mg/mL.

Affinity chromatography was performed using an amylose resin that captures 2 mg/mL of the proteins. The resin was washed with 12 volumes of column buffer (20 mM Tris-HCl pH 7.4, 200 mM of NaCl, and 1 mM of EDTA). Both MBP/exochitinase (MBP/exo) and MBP/endochitinase (MBP/endo) linked to the maltose were then eluted using column buffer with the addition of 10 mM maltose. Eluted fractions with a final volume of 1 mL were then quantified with Bradford's method, yielding concentrations of 0.5-2 mg/mL, which were afterward visualized by SDS-PAGE (Laemmli 1970).

To determine if the MBP/exo and MBP/endo had the complete and active chitinase protein, they were cleaved using the Factor Xa protease (New England Biolabs). For cleavage, to a 100 μL of an affinity chromatography fraction at a concentration of 1 mg/mL was added 2 μL of Factor Xa at 0.2 mg/mL, and 1 μL of SDS at 0.5% and were shaken for 16-18 h at 24°C and 80 rpm. MBP-paramyosin-Dsal (New England Biolabs) was used as the cleavage control (New England Biolabs 2003). Afterward, the cleaved sample was concentrated using a centriprep YM-10 Centrifugal Filter Unit (Millipore. Bedford, MA, USA) to a volume of 5 mL with a concentration of 39 mg/mL. The cleaved fusion protein was dialyzed with buffer containing 20 mM Tris-HCl and 25 mM NaCl, pH 8.0 (2 or 3 changes of 100 volumes every 2 hours) and then subjected to ion-exchange chromatography in order to obtain the pure exochitinase or endochitinase protein. The eluted chromatography fractions were then quantified in a spectrophotometer with Bradford's method and visualized by SDS-PAGE (Laemmli 1970).

### Chitinolytic activity

The activity of exochitinase and endochitinase was assessed both qualitatively and quantitatively. The qualitative assessment was performed with 7 μg/μL of the following fractions: -the raw induced MBP/exo and MBP/endo fractions obtained from the sonication of bacteria, -the semipure MBP/exo fraction eluted from affinity chromatography, -the MBP/exo cleavage reaction with Factor Xa, and -the exochitinase obtained from MBP/exo cleavaged and separated using ion-exchange chromatography and -the MBP. β-N-Acetylglucosaminidase (Sigma-Aldrich, St Louis, MO, USA) was used as a positive control. As substrate, 4-methylumbelliferyl-β-D-N,N'-diacetylchitobioside (Sigma-Aldrich) was used dissolved in sodium acetate buffer pH 5.0 (100 mM) at 2.8 or 3.8 μg. The samples were incubated at 32°C for 30 min with constant shaking at 70 rpm. The reaction was monitored by fluorescence emission using a UV lamp.

The enzymatic activity of the MBP/exo and MBP/endo was evaluated using 4-methylumbelliferyl-β-D-N,N'-diacetylchitobioside. A 10 μL of each sample: -MBP/exo, -MBP/endo or a commercial chitinase from *Trichoderma viride *(Sigma-C8241) (Sigma-Aldrich) at a concentration of 0.005 μg/μL, and 90 μL of sodium acetate buffer pH 6.0 (10% Triton X-100 and 10 mM 2-mercaptoethanol) was mixed with 40 μL of the substrate (1 mg of 4-methylumbelliferyl-β-D-N,N'-diacetylchitobioside in 3.5 mL of 100 mM sodium acetate buffer pH 5.0). After incubation at 27°C for 5, 10, 15, 20 and 25 min the reaction was stopped by mixing an aliquot (20 μL) of the reaction mixture with 180 μL of 0.2 M Na_2_CO_3_. Fluorescence was measured with a Fluoroskan fluorometer (excitation: 360/40 nm; emission: 460/40 nm). A standard curve was calculated based on the fluorescence of 4-methylumbelliferone (0, 5, 10, 15, 20, 25, 30, and 50 mM) dissolved in 0.2 M Na_2_CO_3_. Protein concentration was quantified with Bradford's method, which used different bovine serum albumin (BSA) dilutions in sodium acetate buffer pH 6.0. Activity slopes (nM 4-methylumbelliferyl/min) were determined for each protein, and the value of nM methylumbelliferyl/min was expressed relative to the amount of protein present (nM 4-methylumbelliferyl/min/μg of protein).

### Effect of chitinolytic proteins incorporated in diets against the coffee berry borer larvae

To determine the effect of the exochitinase and the endochitinase on the coffee berry borer, 200 mL of the meridic coffee-based diet, containing 26.6 g of ground parchment coffee with 47% humidity was prepared (Portilla 1999). From this, 5 mL was taken and mixed with 3.36 mg of the proteins to be evaluated, diluted in 700 μL of water to yield a concentration of 0.5% (w/w). The diets containing each individual protein and the control without protein were placed on a 96-well ELISA plate, and 25 wells were filled with ~200 μL of the diet for each treatment. The diet was allowed to dry to 58-60% moisture content, and each well containing the diet was infested with two coffee berry borer eggs and placed in the incubation room at 26°C and ± 75% humidity. The numbers of live and dead insects were recorded daily along with their developmental stages.

Previously, the fractions eluted by affinity chromatography containing the MBP/exo or MBP/endo were dialyzed against water to decrease the salt concentration, then frozen and lyophilized. Three assays were done: Assay 1 included the following treatments: -MBP/exo protein, -Bovine Serum Albumin **(**BSA) (Bio-Rad), -MBP (New England BioLabs), and -distilled water without protein. Assay 2 included -MBP/exo protein and, - distilled water without protein. Assay 3 included: -MBP/endo protein and, -distilled water without protein. All the assays were performed in duplicate.

### In vitro effect of chitinolytic proteins on *H. vastatrix*

For this test, 30 glass slides 76 mm long × 26 mm wide were covered with Parafilm. Then, one drop (5 μl each) of a urediniospore suspension (86,000 spores ml^-1^) of a *H. vastatrix *isolate compatible with the BI625 genotype of *C. arabica *was added to each half of the slide after being previously subjected to ultrasonication for 30 s at 60 MHz to allow for dissociation of the spores.

Each slide was placed on a petri plate, 15 cm in diameter and 1 cm in height that contained a paper towel saturated with 10 ml of distilled water (humid chamber). These chambers were maintained in the dark at a temperature of approximately 25°C for 6 h to allow for the development of germination tubes. Subsequently, 12 μl of sterile distilled water was added to one of the drops on each plate (control treatment), while to the corresponding paired drop, 12 μl of one of the experimental substances was added (10 slides per treatment) as follows: -MBP/endo protein (5 mg ml^-1^), -MBP/exo protein (6 mg ml^-1^), and bovine serum albumin (BSA) at a concentration of 5 mg ml^-1^.

The plates were then maintained under dark conditions at 22°C for 18 h, at which time a volume of 5 μl of lactophenol cotton blue stain was added to each drop to dye the urediniospores and germ tubes that had been able to form. Treatments were documented with an Nikon Eclipse 90i digital camera under 100X and 400X in a Vosskühler VDS microscope.

### Statistical analysis

#### Coffee berry borer assays

The response variable for the insect bioassays on the artificial diets was the number of live and dead insects recorded daily, for a period of 35 days, along with the number of developmental stages of these insects present in each treatment. The mean number of live insects for each treatment in each assay was then calculated. The effects of the treatments were determined using analysis of variance (ANOVA) according to the analysis model for a completely randomized design at a 5% significance level.

#### Coffee rust assay

The response variable was the percentage of affected germ tubes in the treatments with respect to the number of germ tubes present in water alone. Germ tubes either broken or shrunken were counted as affected tubes. Empty urediniospores without germ tubes, as observed under standard lactophenol cotton blue staining, were considered as the number of lost tubes. A total of 100 spores per drop of each treatment were tabulated.

To determine whether there were significant differences in the percentage of germ tubes affected or lost between treatments, an analysis of variance (ANOVA) test was performed for a completely randomized design with a significance level set at 5%. To determine the treatment with the lowest average of germinated spores, a Tukey test was performed at a significance level of 5%.

## Results

Based on the DNA and predicted protein sequences analysis of both enzymes, 384 aa (out of 655) of the endochitinase protein showed 95% identity and E value = 0 to a segment of the C terminal region of Chitinase C from *Streptomyces albus *J1074 (Accession number D6B6V8, Uniprot). Meanwhile, 320 aa (out of 345) of the exochitinase exhibited 72% identity with E value of 1.0 × 10 -^117 ^to a portion of a putative Chitinase A (Accession number F3ZBD7, Uniprot) from *Streptomyces sp*. Tu6071.

### Expression and production of the MBP/exochitinase (MBP/exo) and MBP/endochitinase (MBP/endo) proteins

**Figure 1:**
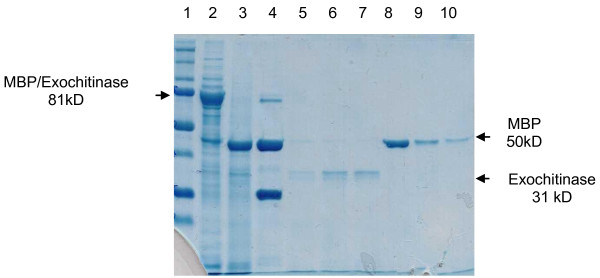
**Exochitinase protein fractions on SDS-PAGE in 4-10% polyacrylamide. Lane 1**. Bio-Rad dual molecular weight marker (range 250-10 kDa). **Lane 2**. Raw induced MBP/exochitinase (81 kDa) obtained by sonication of the bacteria. **Lane 3**. MBP/exochitinase cleaving reaction with Factor Xa protease. MBP (50 kDa) and exochitinase (31 kDa) **Lane 4**. Cleavaging control (MBP/paramyosin) with factor Xa. Paramyosin (27.7 kDa). **Lanes 5, 6, and 7**. Fractions of pure exochitinase obtained from the cleavage of MBP/exochitinase and separated by ion-exchange chromatography. **Lanes 8, 9, and 10**. Fractions of MBP alone obtained from the cleavage of MBP/exochitinase and separated by ion-exchange chromatography.

**Figure 2:**
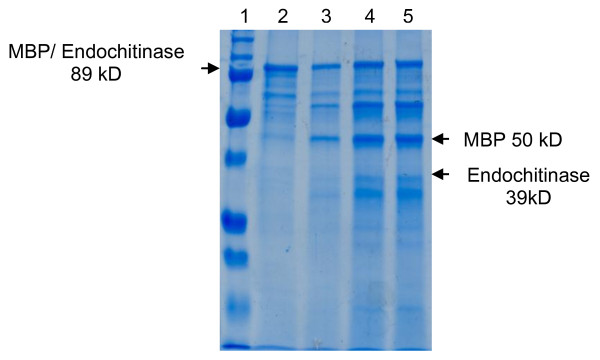
**Endochitinase protein fractions on SDS-PAGE in 4-10% polyacrylamide. Lane 1**. Bio-Rad dual molecular weight marker (range 250-10 kDa). **Lane 2**. Raw induced MBP/endoquitinase (89 kDa) obtained by sonication of the bacteria. **Lane 3, 4 and 5 **MBP/endoquitinase cleaving reaction with Xa factor.

The transformed *E. coli *cells produced the MBP/exo protein, with a size of 81 kDa, and the MBP/endo, with 89.5 kDa, which were purified using affinity chromatography. The MBP/exo protein obtained from chromatography is displayed in Figure 1. The MBP/endo protein is showed in Figure 2.

Quantification using Bradford's method indicated that concentration after affinity chromatography was 0.5-3 mg/mL for both the MBP/exo and MBP/endo.

The MBP/complex permitted the separation of the exochitinase from the MBP protein after cleavage (Figure 1, lane 3), with sizes of 31 kDa, and 50 kDa respectively. Lanes 5, 6, and 7 show the exochitinase protein after being cleaved and purified by ion-exchange chromatography, although at relatively low quantities (0.081 mg/mL).

MBP/endo was also cleaved (Figure 2 lane 3, 4 and 5), resulting in an MBP (50 kDa) protein separated from endochitinase (39 kDa) protein. Again, the amount of total endochitinase in this case was very low.

### Chitinolytic activity

**Figure 3:**
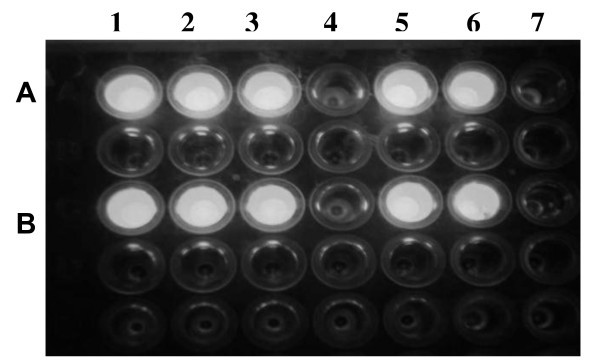
**Qualitative assay of exochitinase activity. Row A**: Substrate: 4-methylumbelliferyl β-D-N,N'-diacetylchitobioside 2.8 μg total and **Row B **Substrate: 4-methylumbelliferyl β-D-N,N'-diacetylchitobioside 3.8 μg total. **Lane 1**. Enzyme: Induced MBP/exochitinase. **Lane 2**. Enzyme: MBP/exochitinase affinity chromatography fraction. **Lane 3**. Enzyme: MBP/exochitinase cleavage. **Lane 4**. Negative control: MBP eluted from ion-exchange chromatography. **Lane 5**. Enzyme: exochitinase eluted from ion-exchange chromatography after been cleaved from a MBP/exochitinase. **Lane 6**. Positive control: Enzyme N-acetyl-β-D-glucosaminidase. **Lane 7**. Substrate alone.

The results from the exochitinase qualitative assays (Figure 3) and endochitinase (not shown) indicated that all of the fractions exhibited chitinolytic enzymatic activity. The only protein without activity was MBP. The results from the quantitative assay showed that the commercial chitinase had an activity of 10.5 nM MU/min/mg of protein, while the activity in the MBP/exo was 5.3 MU/min/μg of protein and in the MBP/endo was 0.7 MU/min/μg.

### Assaying of effect of exochitinase incorporated in diets against the coffee berry borer larvae

Both the MBP/exo and MBP/endo proteins purified by affinity chromatography were used to determine the effect of exochitinase on the coffee berry borer since they showed chitinolytic activity even when bound to MBP. The recombinant proteins could be obtained in considerable amounts in this system and MBP alone was assayed as a negative control to determine any biological activity of this protein on the coffee borer.

**Figure 4:**
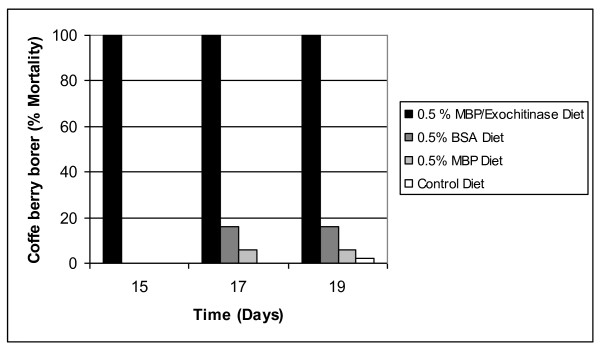
**Effects of the MBP/exochitinase, MBP, and BSA at 0.5% (w/w) assayed in artificial coffee-based diets on the mortality of coffee berry borer larvae**.

In the diet with MBP/exo, a delay in larval growth was observed along with 100% mortality at 15 days in comparison to the diets that had MBP or BSA, which both showed a mortality percentage of less than 10% at the end of the experiment (Figure 4). The insects on the control diet with water presented mortalities of less than 2%, and all insects that survived in this group reached the adult stage at 22-25 days. ANOVA showed significant differences among the mean mortality values under different treatments (F = 8.4; *p *< 0.05), with higher mortality on the MBP/exo diet. There were no differences among treatments with BSA, MBP, and water.

**Figure 5:**
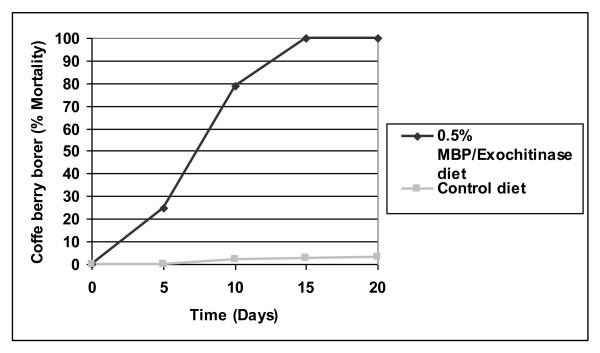
**Effect of the MBP/exochitinase at 0.5% (w/w) assayed in artificial coffee-based diets on the mortality of coffee berry borer larvae**.

**Figure 6:**
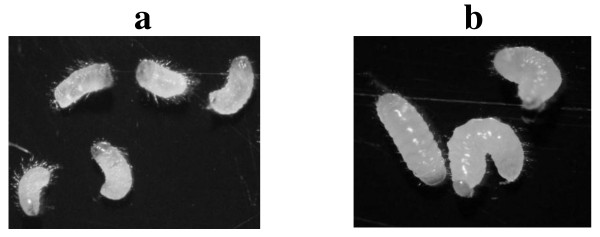
**stages of coffee berry borer larvae consuming artificial diets with and without the MBP/exochitinase. a**. State of the insects fed on a diet with addition of 0.5% (w/w) MBP/exochitinase. The picture shows the maximum size reached by the larvae after all of them had died at 15 days of evaluation. **b**. State of the insects fed on a control diet (15 days). These larvae developed normally and then reached the adult stage.

The second assay of the diet with MBP/exo (Figure 5) showed that, after eggs eclosion in the first days, the *H. hampei *larvae were active and feeding. Since the third day, a delay in the development and growth of the insects could be seen, with fewer larvae with smaller sizes relative to larvae on the control diet (Figure 6). At 15 days of testing, the MBP/exo diet caused a mortality of 100% compared to the control diet, where the mortality did not surpass 10%. This difference was statistically significant.

**Figure 7:**
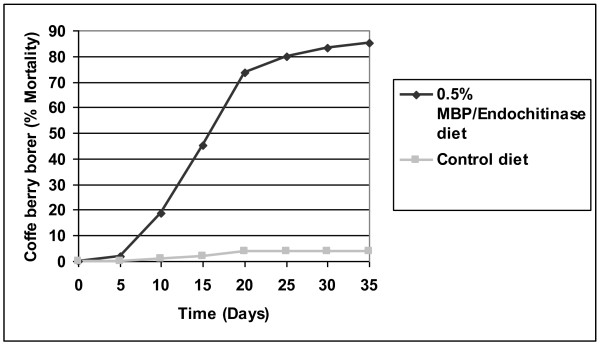
**Effect of the MBP/endochitinase at 0.5% (w/w) assayed in artificial coffee-based diets on the mortality of coffee berry borer larvae**.

The effect of the MBP/endo is observed in Figure 7, in which the protein showed effect on the larvae with delay in growth and 84% mortality at day 30 of the assay. The insects that survived the treatment with MBP/endo remained in the larval stage until day 35, when the observations were stopped. By contrast, all insects on the control diet reached the adult stage by day 25.

### In vitro effect of chitinolytic proteins on the coffee rust fungus

**Figure 8:**
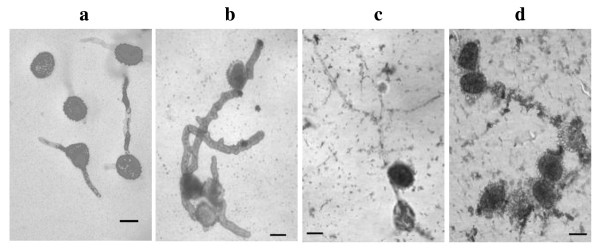
**Effect of Chitinolytic enzymes on the structure germ tubes of H. vastatrix after 18 h of treatment application**. MBP (Maltose binding protein). Germinating uredospores treated with a. water, showing normal growth; b. BSA (Bovine Serum Albumin) displaying increased cell wall thickness; c. MBP/endochitinase and d. MBP/exochitinase, resulting in germ tubes either broken or shrunken. (Bar = 20 μm).

The control treatment (sterile distilled water) did not affect the internal content of the urediniospores and their emerging germ tubes. Germination behavior was similar to the control under the BSA treatment, although a thickening of the cell walls was notorious along the germ tubes (Figure 8). For treatments involving recombinant MBP/exo and MBP/endo proteins, the internal content of the germinated spores was empty. Additionally, clusters or fragments that had been part of the degraded walls of the spores or the germ tubes were observed (Figure 8). This damage depended on the treatment applied to the urediniospores (p < 0.0001). According to the Tukey test at a 5% level of significance, MBP/exo and MBP/endo proteins had a negative structural effect on the germ tube, while treatments with water or BSA did not cause any damage to these structures (Table 1).

**Table 1:** Effect of chitinolytic enzymes on the integrity of germ tubes from *Hemileia vastatrix *urediniospores

Treatment		$\bar{X}$**%**	SE
MBP-Chitobiosidase	A*	94.0	2.79

MBP-Endochitinase	B	82.3	4.36

BSA	C	1.10	0.75
Distilled water	C	0.0	0.0

*Differing letters indicate significant difference in means according to the Tukey test at a 5% level.

X. Average percentage of rust urediniospore germ tubes affected.

SE. Standard error for each of the applied treatments.

## Discussion

Historically, control of insect pests and diseases in commercial crops has been predominantly based on the use of pesticides. Current environmental trends and developments restrain the use of synthetic chemicals and procure the application of alternatives that complement an integrated pest management approach that also includes genetic, biological and cultural control measures. This is especially true in semi-perennial agricultural systems like coffee, where it is common to find plantations over 20 years old, closely associated to a diverse combination of plant, animal and microbial species. The identification of proteins that could be introduced in semi-perennial crops to generate insect and pathogen resistant plants is important for the sustainability of the agronomical production. Because of their target, chitinases are suitable candidates among those proteins.

The goal of the study was to produce pure chitinase proteins to assay them in artificial diets of coffee berry borer larvae, and in vitro assays of coffee rust. However, due to the low efficiency of MBP cleavage with the Factor Xa (Figures 1 and 2), that resulted in insufficient amounts of pure exochitinase and endochitinase proteins, it was decided to test instead the MBP/exo and MBP/endo fusion proteins for chitinase and biological activity. The results indicate that the pMAL ™ protein expression system enables the production of the exochitinase and endochitinase in such a way that they maintain their enzymatic activity even when bound to MBP. Besides, this study confirmed that the MBP by itself did not interfere with the chitinase enzymatic activity of the protein neither affected the development of the insects nor the fungi, therefore permitting the evaluation of the chitinase- MBP fusions for their enzymatic and biological activities.

The effect of feeding larvae on diets amended with MBP/exo or MBP/endo proteins was insect mortality and growth delay, confirming the results obtained by Broadway et al. (1998) on *H. hampei*, who reported 100% insect mortality after 30 days of consumption of a 1% (w/w) mix of chitinolytic enzymes, but improving the efficiency in the case of MBP/exo by reducing the time for 100% mortality to 15 days, with only half the amount of protein, 0.5% (w/w). Meanwhile the MBP/endo exhibited a lower performance, causing 85% mortality after 35 days.

Considering that the target site for both enzymes is the peritrophic membrane on the insect digestive tract, as previously reported on *Trichoplusia ni *(Góngora et al. 2001), the greater activity observed in MBP/exo might have to do with either intrinsic affinity for the substrate, increased hydrolytic activity or the particular spatial availability of the chitin substrate on the surface of the coffee berry borer intestine.

Although other proteins have been tested for their inhibiting effects on the coffee berry borer, the great majority have been assayed by isolating the proteins from the intestine of the borer and testing whether their in vitro activity can be reduced by the tested substances. Efficiency on live insects remains to be confirmed for borer control. For instance, an aspartic protease inhibitor from *Lupinus bogotensis *(Molina et al. 2010), proved to be effective against the intestinal aspartic proteases of *H. hampei*, and the α-amylase inhibitor gene α-AI1 from *Phaseolus vulgaris*, transformed into *C. arabica *plants by Barbosa et al. (2011), showed inhibition of *H. hampei *α-amylase enzyme activity assayed in vitro using extracts of transgenic seeds. One of the limitations to assaying coffee berry borer inhibitors in vivo is the need for a large quantity of protein. Padilla et al. (2006) assayed an extract of *Brachiaria decumbens *in artificial diets in which an α-amylase inhibitor caused coffee borer mortality of 40 and 95% at 12 and 24 days, respectively, and López-Pazos et al. (2009) investigated the effects of recombinant Cry1B and Cry3A proteins from *Bacillus thuringiensis *in artificial diets on *H. hampei*. These proteins yielded mortalities of 20 to 45% in larvae after 6 days of observation. Together with chitinases, these proteins could be used in a multi-partite approach to confer insect resistance to coffee plants.

The antifungal effect of chitinolytic enzymes has been described in multiple studies (Broadway et al. 1995; Govinda et al. 2011; Lorito et al. 1993; Limón and Benítez 2002, 2004). When chitinases hydrolyze the chitin polymer, the cell wall is affected in its fundamental structure, which in turn influences critical physiological processes in germination, cell division, and elongation of the germ tube (Gooday et al. 1992; Seidl 2008), and can disturb activities directly related to pathogenesis, since the germ tube allows the penetration and colonization of the fugus into plant tissues, and intercellular mycelia parasite its cells. Nevertheless, in the coffee leaf rust interaction, the activation of plant chitinases has been reported in both resistant and susceptible plants (Maxemiuc-Naccache et al. 1992; Guerra-Guimarães et al. 2009), leaving unclear the possible role of these enzymes in the defense mechanisms against *H. vastatrix*. The present study, however, presents the first evidence of a chitinolytic effect on *H. vastatrix*, affecting the germ tubes of this fungus in vitro.

The quantity of chitinolytic protein sufficient to obtain antifungal activity has been observed to be extremely variable. It seems to be closely related to the organism it is derived from, its purity, the specific activity and the characteristics of the targeted fungus. For example, while Deng et al. (2007) used 0.7 mg ml^-1 ^of a *Trichoderma atroviride *endochitinase for a 100% inhibition of *Penicillium digitatum*. Broadway et al. (1995) used a 6 to 20 fold less concentrated mixture of *S. albidoflavus *exochitinases to inhibit *Botrytis cinerea *and *Fusarium oxysporum*. In contrast, a higher concentration was used in this exploratory phase of recombinant *S. albidoflavus *chitinases since only an affinity chromatograpy purification protocol was applied to the protein extracts and the protein still remained bound to MBP. Taking into account the biological activity observed lower protein concentrations should be tested.

Chitinolytic enzymes, upon introduction into a plant heterologous system, can generate protection or defense against fungal attacks, as observed in tobacco and potato plants modified to generate resistance to *Rhizoctonia solani, Botrytis cinerea, Alternaria alternata*, and *A. solani *(Lorito et al. 1993, Dana et al. 2006), in cotton plants with resistance to *R. solani *and to *A. alternata *(Chandrakanth et al. 2003), and in plants such as apple trees (Bolar et al. 2001), broccoli (Mora and Earle 2001), or the lemon tree (Distefano et al. 2008) to generate resistance to other pathogenic fungi. Similarly, alternative chitinases have been tested to provide protection against insects (Góngora et al. 2001, Ding et al. 1998; Kramer and Muthukrishnan 1997). However, no assays have been conducted to date using the same set of enzymes against two broadly different groups of pests.

This study identified two chitinolytic proteins with significant biological activity against two of the main coffee limiting pests, the coffee berry borer and the coffee leaf rust, therefore making the encoding genes appropriate candidates to be further exploited in coffee and other plants in order to introduce a wide genetic resistance and to reduce the participation of chemical control in the integrated management of plantations.

## Competing interests

The authors declare that they have no competing interests.
